# Disparities in Risk Perception: A Comparison Between Medical and Non-medical Professionals Using Propensity Score Matching

**DOI:** 10.7759/cureus.84718

**Published:** 2025-05-24

**Authors:** Naomi Akiyama, Shihoko Kajiwara, Nagisa Adachi, Tomoya Akiyama

**Affiliations:** 1 Graduate School of Nursing, Nagoya City University, Nagoya, JPN; 2 School of Nursing, Gifu University of Health Science, Gifu, JPN; 3 Center for Postgraduate Clinical Training and Career Development, Nagoya University Hospital, Nagoya, JPN

**Keywords:** hospitalization, infection, non-medical professional, nurse, nursing staffing, propensity score matching, risk perception

## Abstract

Introduction

Disparities in risk perception between non-medical professionals and nurses can affect interdisciplinary communication and decision-making in healthcare. In this study, we explored these differences with a focus on the risks during hospitalization, infection risks, and family accompaniment to inform strategies for improving patient-centered care.

Methods

This cross-sectional study was conducted using a propensity score-matched sample of 826 participants in Japan, including 413 non-medical professionals and 413 nurses. A structured questionnaire was used to assess perceptions of risks related to falls, pressure ulcers, delirium, infection, and family accompaniment during hospitalization. Factor analysis was used to evaluate the validity and reliability of the scale, and t-tests were used to compare group differences.

Results

The developed scale demonstrated a cumulative contribution rate of 74%, with good internal consistency (Cronbach's alpha = 0.892 for the first factor). Significant differences were observed between the groups for all parameters (p < 0.001). Non-medical professionals exhibited higher risk perception scores for falls, pressure ulcers, and infections, reflecting a lower tolerance for clinical risks than nurses. Conversely, nurses displayed greater acceptance of uncertainty and family accompaniment practices.

Conclusions

This study highlights the persistent differences in risk perceptions between non-medical professionals and nurses and emphasizes the need for interventions to bridge these gaps. Addressing the mechanisms of uncertainty and distress and fostering interdisciplinary communication is essential for improving collaborative care and patient outcomes. In the future, researchers should explore the factors driving these differences and develop strategies to align the perceptions of healthcare stakeholders.

## Introduction

Medical care is inherently affected by various forms of uncertainty, including ambiguities in diagnosis, treatment, and patient experiences [1-3]. Previous studies have shown that non-medical professionals often exhibit lower tolerance for such uncertainties compared with medical professionals, leading to potential communication issues [4,5]. In addition, fragmented care, especially among patients with severe chronic illnesses, emergency cases, and older adults, frequently leads to information gaps, defined as breakdowns in the communication or the transfer of critical clinical information [6]. In previous research, the authors have emphasized that these gaps can lead to delayed diagnoses, repeated testing, and poor patient outcomes, particularly in emergency care settings, where time-sensitive decisions are crucial [7]. Furthermore, the effective use of health information technology, such as electronic medical records, has been shown to reduce these gaps by facilitating better coordination among healthcare teams [8]. Documentation in healthcare often reflects the perspective of a medical professional, which may inadvertently exclude the nuances of the patient’s experience. If healthcare providers fail to recognize the gap between their perspective and that of the patient, this disconnect is unlikely to be captured in the records, potentially perpetuating disparities in understanding and delivering care.

In Japan, the establishment of medical accident investigations and support centers aims to mitigate medical accidents by analyzing cases and disseminating findings nationwide. Consultations with these centers have steadily increased, with 2023 showing a notable increase compared with 2022. Intriguingly, a considerable portion of these inquiries, particularly from bereaved families, centers on determining whether their cases qualify as medical accidents. Of the 1,213 consultation cases from bereaved families, 896 specifically sought clarification on this issue [9]. Typically, such questions are addressed directly by medical institutions. However, reliance on these centers suggests a perceptual gap between the public and medical professionals, even in critical situations such as medical accidents. This study aimed to 1) examine differences between medical and non-medical professionals in their perceptions of uncertain clinical situations, focusing on specific risks, such as falls, pressure ulcers, infections, and family accompaniment, and 2) validate these disparities using observational data analyzed through propensity score matching.

This study aimed to 1) examine differences between medical and non-medical professionals in their perceptions of uncertain clinical situations, focusing on specific risks such as fall, pressure ulcers, infections, and family accompaniment, and 2) validate these disparities using observational data analyzed through propensity score matching.

Hypothesis

A hypothesis of this study is that non-medical professionals perceive greater staffing needs than medical professionals, which may influence collaborative decision-making and the quality of patient care.

## Materials and methods

We employed a cross-sectional online survey to examine the perceptions of uncertainty in medical care among individuals in Japan. Data were collected using an Internet-based platform provided by the survey company. Participants were divided into two groups: non-medical professionals and nurses, who were selected on the basis of predefined criteria.

Participant selection

The non-medical professional group included individuals with no professional experience in the healthcare field, whereas the nursing group comprised licensed professionals actively engaged in nursing practice. The sample consisted of adults aged 20 years and older residing in Japan. Participants were randomly sampled from among the monitors of the Internet survey company (Cross Marketing Inc.) and stratified according to age, sex (in similar proportions), and residential area. Regarding residential areas, the number of participants selected from the monitors was determined according to the prefecture’s population composition ratio. The Internet survey company sent a questionnaire to all participants and collected questionnaire responses until the target number was reached. The Internet survey company sent a questionnaire to all participants and collected questionnaire responses until the target number was reached. To ensure data quality, Cross Marketing Inc. implemented measures to prevent duplicate or fraudulent responses. Checks for duplicate registrations were conducted at the time of participant registration and during regular data cleaning processes, using personal demographic information. Participants identified as duplicates or exhibiting irregular response patterns were removed from the active survey pool. In addition, if multiple responses were detected from the same IP address, such responses were excluded before the final dataset was delivered. The population composition was published by the Statistics Bureau of the Ministry of Internal Affairs and Communications [10].

Questionnaires were sent to the selected participants, and responses were collected until the desired sample size was achieved. To maintain a 6.5% margin of error at the 95% confidence interval, 228 participants were required for each age and sex category. Considering potentially invalid responses, the target sample size was set at 1,500 participants. In terms of the nurse group, the sex distribution among Japanese nurses is highly skewed, with 91.9% female and 8.1% male nurses [11]. Therefore, the nurses were randomly sampled and stratified according to age. In particular, 228 participants were targeted for the 40-59-year age group, while fewer participants with a greater margin of error were targeted for other age groups, reflecting the availability of nurses as reported in a 2020 survey on the age distribution of employed nurses [11]. Because invalid responses from medical professionals were considered unlikely, the total target number of nurses was set to 600.

Among the 1,500 non-medical professionals, 70 held medical qualifications and were excluded from the study, as they were considered to understand medical practices and therefore did not qualify as non-medical professionals. Ultimately, the study included 600 nurses and 1470 non-medical professionals.

Survey design and data collection

To ensure internal validity in developing questions about the five-nurse staff, nursing administrators and researchers collaborated to review and refine the content. A preliminary study was then conducted with five nurses, and on the basis of their feedback, the final set of seven questions was determined. We developed original questionnaire items to assess perception of uncertainty in medical care, with a focus on two domains: (1) five items focusing on “awareness and acceptance of risk and restrictions during hospitalization,” such as infection transmission, pressure ulcer development, falls, disorientation, use of physical restraints, and (2) two items addressing “family support for a child or elderly hospitalizations.” Full survey items are provided in the Appendix.

The structured questionnaire also included a section evaluating perceptions of uncertainty in medical care, with responses measured on a seven-point Likert scale (1 = strongly agree, 7 = strongly disagree).

Data analysis

First, a factor analysis with varimax rotation was conducted to explore and confirm the underlying structure of the scale and evaluate its construct validity. To examine the reliability of the seven-item questionnaire, we used data from 2,030 participants and calculated the Cronbach's alpha coefficients. 

Second, propensity score matching was used to sample participants from the non-healthcare population, ensuring that they were matched one-to-one with nurse participants on the basis of relevant covariates. The covariates used for matching included age, sex, education level, marital status, child status, population size in the living area, and medical care user. The total score for the primary factor, “perception of risk hazard,” was dichotomized using the median value as the cutoff point. Logistic matching was conducted using a caliper coefficient of 0.05. This method ensured that the matching process effectively reduced selection bias and facilitated a balanced comparison between groups. After propensity score matching, we assessed covariate balance between nurse participants and non-healthcare participants by comparing demographic variables using the χ2 test or Fisher's exact test, as appropriate. In addition, the balance between groups was assessed using the standard mean difference (std difference). The same tests were also applied to the unmatched sample for reference. This process was used to verify whether the matching successfully reduced baseline differences between the groups. Following propensity score matching, the Kruskal-Wallis test was used to compare outcomes between nurses and non-medical professionals in the matched sample. For reference, similar comparisons were performed between nurses and non-medical professionals in the unmatched sample as well.

All statistical analyses were conducted using JMP, version 17.0 (SAS Institute Inc., Cary, NC, USA). A two-sided p-value of less than 0.05 was considered statistically significant.

Ethical consideration

This study was conducted with the approval of the ethics committee of Gifu University of Health Science (approval number: 2022_19). All the participants provided informed consent, and the data were collected anonymously.

## Results

Study design

This study employed a cross-sectional design using an Internet-based survey to collect data from participants. The survey was conducted at a single point to examine the variables of interest.

Participant flow diagram

**Figure 1:**
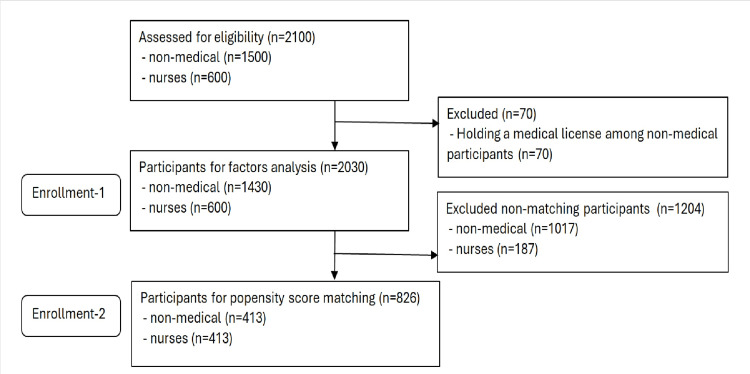
Participant flow diagram.

Figure 1 shows the STROBE flow chart. A total of 2,100 individuals were assessed for eligibility, including 1,500 non-medical participants and 600 nurses. During the initial screening, 70 participants from the non-medical group were excluded because they held medical licenses. After this exclusion, 2,030 participants (1,430 non-medical participants and 600 nurses, Enrollment 1) were included in the first phase of factor analysis.

Subsequently, propensity score matching was performed to balance the characteristics of the two groups. Seven factors assumed to be related to the primary outcome - age group, sex, education level, marital status, child status, population size in the living area, and medical care user - were included as matching variables. During this process, 1,204 participants were excluded owing to non-matching scores; they comprised 1,017 non-medical participants and 187 nurses.

The final sample for the propensity score-matched analysis included 826 participants, comprising 413 from the non-medical group and 413 nurses (Enrollment 2).

Characteristics

Table 1 shows the characteristics of the non-medical professionals and nurses in both the matched and unmatched groups. In the "All" group, the most prevalent age group was 40-59 years, accounting for 36.7% of participants. Females constituted the majority (59.6 %) of the participants. In terms of educational level, university or graduate school attainment was the highest (40.6%). Most participants were married (65.6%), and slightly more than half (52.2%) had children. When considering the population size of living areas, the largest proportion of participants (34.8%) resided in capital areas. Finally, most participants (64.5%) reported that they were not using medical care services.

**Table 1:** Participant characteristics (Enrollment 1). a: χ^2^ test, b: Fisher's exact test

	All	Non-medical	Nurses	p value
	n=2030	n=1430	n=600	
Age				
20-39 years	670(33.0)	200(33.3)	470(32.9)	<0.001^a^
40-59 years	745(36.7)	271(45.2)	474(33.2)	
60 years and older	615(30.3)	129(21.5)	486(34.0)	
Sex				
Men	820(40.4)	95(15.8)	725(50.7)	<0.001^b^
Female	1210(59.6)	505(84.2)	705(49.3)	
Education level				
Unievrsity/graduate school	825(40.6)	161(26.8)	664(46.4)	<0.001^a^
Vocational school/ junior college	726(35.8)	431(71.8)	295(20.6)	
Junior high school/ high school	479(23.6)	8(1.3)	471(32.9)	
Marital status				
Unmarried	698(34.4)	163(27.2)	535(37.4)	<0.001^b^
Married	1332(65.6)	437(72.8)	895(62.6)	
Child status				
Without children	970(47.8)	238(39.7)	732(51.2)	<0.001^b^
Having children	1060(52.2)	362(60.3)	698(48.8)	
Population size in the living area				
Capital	706(34.8)	197(32.8)	509(35.6)	0.114^a^
City	472(23.3)	149(24.8)	323(22.6)	
Middle city	332(16.4)	87(14.5)	245(17.1)	
Town	410(20.2)	138(23.0)	272(19.0)	
Small town	110(5.4)	29(4.8)	81(5.7)	
Medical care user				
Yes	721(35.5)	220(36.7)	501(35.0)	0.509^b^
No	1309(64.5)	380(63.3)	929(65.0)	

Factor analysis results: awareness of the risk and care influenced by nurses’ staffing scale

The participants in this study are those indicated as Enrollment 1 in Figure 1, with data from a total of 2,030 individuals (1,430 non-medical participants and 600 nurses).

A factor analysis with varimax rotation was conducted for the seven items. However, one item, related to restraint (“I think it is acceptable to use measures such as wearing mittens or restraining my hands to prevent me from unintentionally removing important IV tubes during hospitalization, even if it restricts my freedom of movement”) showed poor factor loadings and was subsequently excluded. A second factor analysis was performed using the six items. Three distinct factors were identified through varimax rotation.

Table 2 presents the factor analysis results, including the factor loadings for each item across the three identified factors. The first factor, labeled "perception of risk hazards," consisted of items related to falls, bedsores, and delirium, which loaded strongly on this factor, reflecting its association with perceived risks during hospitalization. The second factor, labeled "perception of accompaniment," included items related to the accompaniment of older and child patients, highlighting the perception of family presence during hospitalization. The third factor, labeled the "perceived risk of infection," was represented by the item regarding the risk of contracting an infectious disease, indicating a distinct dimension of perceived risk. The cumulative contribution rate of these three factors was 74.3%. The standardized Cronbach's alpha coefficients were 0.892 and 0.746 for the first and second factors, respectively, which indicates good internal consistency. The Bartlett-adjusted chi-square value (χ² = 2.293) was small, which suggests that the three-factor model fits the data well. The Akaike Information Criterion (AIC, 2.3) and Bayesian Information Criterion (BIC, 2.3) values are also low, supporting the adequacy of the three-factor model.

**Table 2:** Factor analysis with varimax rotation. Using factor analysis with varimax rotation. SD: standard deviation.

Factor	Item	Mean	SD	First factor	Second Factor	Third Factor	Cumulative contribution rate
Perception of Risk Hazard (α=0.892)	Q1: I do not think it is possible for me to fall or slip from the bed during hospitalization. [Falling]	5.3	1.4	0.914	-0.066	0.197	38.8
	Q2: I do not think it is possible for me to develop bedsores on my buttocks or heels during hospitalization due to prolonged pressure from the bed or rails, causing redness, blisters, or wounds. [Wound]	5.3	1.4	0.789	0	0.327	
	Q3: I do not think it is possible for me to become disoriented about time or place, behave unusually, or act in ways I normally would not during hospitalization. [Delirium]	5.0	1.6	0.776	-0.108	0.165	
Perception of Accompaniment (α=0.746)	Q4: I believe it is natural for family members to accompany older patients during their hospitalization. [Accompaniment of older]	3.8	1.3	-0.058	0.797	-0.089	59.0
	Q5: I believe it is natural for family members to accompany children during their hospitalization. [Accompaniment of children]	4.4	1.4	-0.046	0.748	0.049	
Perceived Risk Infection	Q6: I do not think it is possible for me to contract an infectious disease from another patient during hospitalization. [Infection]	5.5	1.4	0.512	-0.033	0.858	74.3

Baseline characteristics before and after matching

In Table 3, the characteristics of nurses and non-medical professionals are shown separately for both the matched and unmatched groups.

**Table 3:** Comparison of characteristics between non-medical professionals and nurses (Enrollment 2). a: χ^2^ test, b: Fisher's exact test

	Matched group				Unmatched group			
	Non-medical	Nurses	p value	Std difference	Non-medical	Nurses	p value	Std difference
	n=413	n=413			n=1017	n=187		
Age(years)								
20-39 years	143(34.6)	142(34.4)	0.804^a^	0.004	327(32.1)	58(31.1)	<0.001^a^	0.022
40-59 years	167(40.4)	175(42.4)		0.041	307(30.2)	96(51.3)		0.440
60 years and older	103(25.0)	96(23.2)		0.042	383(37.7)	33(17.6)		0.461
Sex								
Men	83(20.1)	95(23.0)	0.352^b^	0.071	642(63.1)	0(0.0)	<0.001^b^	1.845
Female	330(79.9)	318(77.0)		0.071	375(36.9)	187(100.0)		1.845
Education level								
Unievrsity/graduate school	162(39.2)	155(37.5)	0.881^a^	0.035	502(49.4)	6(3.2)	<0.001^a^	1.232
Vocational school/ junior college	243(58.8)	250(60.5)		0.035	52(5.1)	181(96.8)		4.602
Junior high school/ high school	8(2.0)	8(2.0)		0.000	463(45.5)	0(0.0)		1.292
Marital status								
Unmarried	132(32.0)	129(31.2)	0.881^b^	0.017	403(39.6)	34(18.2)	<0.001^b^	0.485
Married	281(68.0)	284(68.8)		0.017	614(60.4)	153(81.8)		0.485
Child status								
Without children	185(44.8)	185(44.8)	1.000^b^	0.000	547(53.8)	53(28.3)	<0.001^b^	0.537
Having children	228(55.2)	228(55.2)		0.000	470(46.2)	134(71.7)		0.537
Population size in the living area								
Capital	137(33.2)	144(34.9)	0.976^a^	0.036	372(36.6)	53(28.3)	<0.001^a^	0.178
City	112(27.1)	110(26.6)		0.011	211(20.7)	39(20.9)		0.005
Middle city	66(16.0)	62(15.0)		0.028	179(17.6)	25(13.3)		0.119
Town	79(19.1)	76(18.4)		0.018	193(19.0)	62(33.2)		0.328
Small town	19(4.6)	21(5.1)		0.023	62(6.1)	8(4.3)		0.081
Medical care user								
Yes	138(33.4)	142(34.4)	0.826^b^	0.021	363(35.7)	78(41.7)	0.118^b^	0.123
No	275(66.6)	271(65.6)		0.021	654(64.3)	109(58.3)		0.123

In the matched group, there were no significant differences between non-medical professionals and nurses in terms of age (p = 0.804), sex distribution (p = 0.352), education level (p = 0.881), marital status (p = 0.881), child status (p = 1.000), population size of living areas (p = 0.976), or medical user (p = 0.826). However, in the unmatched group, these characteristics showed significant differences, including age (p < 0.001), sex distribution (p < 0.001), educational level (p < 0.001), marital status (p < 0.001), child status (p < 0.001), and population size (p < 0.001).

Medical service use did not differ significantly between the groups (matched: p = 0.826; unmatched: p = 0.118).

Standardized mean differences (SMDs) were calculated to evaluate the covariate balance. Several covariates demonstrated significant imbalance prior to matching, with some SMDs exceeding 1.0. Matching substantially improved balance across most covariates, although complete balance could not be achieved for variables with complete or near-complete separation.

Comparison between non-medical professionals and nurses using propensity score matching

Propensity score matching was used to compare risk perception scores between non-healthcare professionals and nurses, as shown in Table 4. Using 826 participants matched through propensity score analysis (Enrollment 2, Figure 1), comparisons between non-medical professionals and nurses were conducted for each item. The results are presented in Table 4. Significant differences were found in all items (Q1-Q6) between the two groups (p < 0.001 for each comparison). For items Q1, Q2, Q3, and Q6, nurses reported higher scores than non-medical professionals did. Conversely, for items Q4, non-medical professionals obtained higher median scores than nurses did.

**Table 4:** Comparison of risk perception scores between non-medical professionals and nurses using propensity score matching. Ranging from 1 (strongly agree) to 7 (strongly disagree) and the 25th–75th percentile range. The Kruskal-Wallis rank-sum test was performed for the analysis.

	All			Mached group			Unmatched group		
	Non-medical	Nurses	p value	Non-medical	Nurses	p value	Non-medical	Nurses	p value
	n=1430	n=600		n=413	n=413		n=1017	n=187	
	Median (Range)	Median (Range)		Median (Range)	Median (Range)		Median (Range)	Median (Range)	
Q1 (Falling)	5(4-6)	6(5-7)	<0.001	5(4-6)	6(5-7)	<0.001	5(4-6)	6(5-7)	<0.001
Q2 (Wound)	5(4-6)	6(5-7)	<0.001	5(4-6)	6(4-7)	<0.001	5(4-6)	6(5-7)	<0.001
Q3 (Delirium)	4(4-5)	6(4-7)	<0.001	4(4-6)	6(4-7)	<0.001	4(4-5)	6(4-7)	<0.001
Q4 (Accompariment of elderly)	4(4-5)	4(4-5)	<0.001	5(4-6)	4(4-5)	<0.001	4(4-5)	4(4-5)	0.056
Q5 (Accompariment of children)	4(4-5)	4(3-4)	<0.001	4(3.5-5)	4(3-4)	<0.001	4(4-5)	4(3-4)	<0.001
Q6 (Infection)	5(4-6)	6(5-7)	<0.001	5(4-7)	6(5-7)	<0.001	5(4-6)	6(5-7)	<0.001
Perception of Risk Hazard	15(12-18)	18(15-21)	<0.001	15(12-18)	18(14-21)	<0.001	15(12-18)	17(15-21)	<0.001
Perception of accompaniment	8(8-10)	8(7-9)	<0.001	8(8-10)	8(7-9)	<0.001	8(8-10)	8(7-9)	<0.001
Perception Risk Infection	5(4-6)	6(5-7)	<0.001	5(4-7)	6(5-7)	<0.001	5(4-6)	6(5-7)	<0.001

## Discussion

Based on the results, differences in the perceptions of indicators related to nurse staffing between non-medical professionals and nurses were identified. The main findings of this study are twofold. First, a scale related to risks and care influenced by nurse staffing was developed. Second, this scale enabled the measurement of differences in perceptions between non-medical professionals and nurses.

Development of the risk and care influenced by the nurse staffing scale

Several review articles have been published regarding the impact of nursing staffing on patient outcomes. While the impact of nurse staffing on patient outcomes is often viewed as limited owing to methodological constraints in existing studies, indicators such as falls, pressure ulcers, and healthcare-associated infections are recognized as areas where nurse staffing levels influence patient outcomes [12-14].

These review articles highlight mortality rates as indicators of how nurse staffing levels affect patient outcomes. However, asking about mortality rates is not considered appropriate for non-medical professionals, and this was, therefore, not addressed in this study. There are elements of uncertainty regarding the incidence rates of pressure ulcers and falls, and these outcomes are not solely determined by nurse staffing levels. Consequently, in recent discussions, they are no longer regarded as primary indicators. Nonetheless, these indicators, which reflect the inherent uncertainties of medical care, are considered items that both medical and non-medical professionals should recognize. A report by Dr. Otaki published on a Japanese website states that 531 medical accidents related to delirium were identified in the Project to Collect Medical Near-Miss/Adverse Event Information records between 2017 and 2021 [15]. Delirium can occur regardless of the nurse staffing level. However, temporary delirium caused by hospitalization or treatment can create a situation that is difficult to understand, not only for patients but also for their family members.

According to a survey conducted by Keeping Moms Smiling, 80-90% of accompanying caregivers were reported to provide a wide range of care, including assistance with meals, toileting, hygiene, medication administration, supervision, putting patients to bed, playtime, and emotional support. Among these, there were instances in which care was typically handled and observed by nursing staff. In Japan, hospital regulations concerning inpatient nursing care stipulate that it is permissible for caregivers to accompany pediatric patients who have difficulty understanding their treatment, provided that they have the approval of a physician. However, 79.1% of respondents (n = 2,596/3,282) indicated that they had been requested by the hospital to stay with the patient during hospitalization, significantly exceeding 20.9% (n = 686/3,282) who reported that they had not been asked to do so [16]. This issue has been widely reported in Japanese society and has become a social problem, with hospitals being criticized for shifting the burden on parents of hospitalized children owing to a shortage of nurses. There are also reports suggesting that children perceive their hospitalization experiences positively, regardless of whether their parents accompany them [17]. While previous studies on issues related to nurse staffing have not highlighted accompaniment as a key factor, it was included in this scale to reflect Japan’s unique circumstances.

One excluded item addressed whether restraints should be used when bed rest is necessary. In Japan, there has been a movement to promote nursing practices that avoid restraint through appropriate nurse staffing and the use of appropriate medications. However, responses to this item varied, likely depending on the perceived risk posed by the patient or the specific treatment situation. Therefore, these items were excluded from the scale.

The scale developed in this study consisted of six items and demonstrated a cumulative contribution rate of 74%. This is considered a moderately effective scale for identifying differences in perceptions related to the allocation of non-medical and medical staff.

Differences in perceptions regarding the allocation of non-medical staff and nurses

The results of this study demonstrate that even after employing propensity score matching to rigorously adjust for confounding individual attributes among participants, significant differences in perceptions of nurse staffing indicators persisted between non-medical and medical professionals. Non-medical professionals exhibited higher risk perception scores for in-hospital risks than did nursing staff. Specifically, non-medical professionals demonstrated a lower tolerance for risks such as falls, pressure ulcers, and delirium than did nurses. Similarly, their acceptance of infection risk was notably lower than that of the nursing professionals.

As suggested in the model of uncertainty distress of Freeston et al. (2020), intolerance of uncertainty comprises dispositional tendencies and situational responses to uncertainty shaped by real-world variables and life disruptions [18]. As frontline healthcare workers, nurses are routinely exposed to clinical risks and uncertainties, which may foster a higher tolerance for uncertainty through professional adaptation. Conversely, non-medical professionals with limited direct exposure to such risks may perceive them as more severely affected and react to heightened distress. Studies examining the differences in risk perception between non-medical and medical professionals are scarce. A report by Listyowardojo et al. is one of the few studies in this area highlighting significant differences in health risk perception between these two groups [19]. Akiyama et al. reported that non-medical patients were less tolerant than nurses and had lower uncertainty scores, which included diagnostic, treatment, or drug uncertainty, and complications [5]. To address the observed differences in risk perception between non-medical professionals and nurses, future research should focus on developing and evaluating educational programs aimed at harmonizing these perceptions.

These findings highlight the importance of understanding the mechanisms that underlie uncertainty and distress in healthcare settings. Bridging the gap in risk perception between non-medical and medical staff is critical for fostering effective collaboration and a unified response to healthcare challenges.

The issue of family accompaniment during the hospitalization of children or older individuals is complex. While hospitals cannot mandate that family members provide accompaniment, cultural norms emphasizing familial caregiving and the context of attachment formation in children often justify this practice. The findings of this study indicate that the general public is more inclined toward this perspective, reflecting a stronger orientation toward family caregiving [16]. In addition, the financial burden of hospitalized children on parents has been documented in Japan. It is essential for healthcare providers to support families in the decision-making process to ensure that choices align with the specific circumstances of both the family and child.

This study underscores that bridging perception gaps regarding healthcare risks is not merely an academic concern but a pressing societal issue. As healthcare systems increasingly emphasize patient-centered care and shared decision-making, fostering mutual understanding between healthcare providers and the public becomes essential. The scale developed in this study offers a practical tool for identifying and addressing these perceptual gaps, ultimately contributing to safer, more collaborative healthcare environments.

Limitations

This study has two primary limitations: The first is the cumulative contribution rate of the scale. The developed scale’s cumulative contribution rate is limited to 74%. Developing a more precise scale remains a challenge. Freeston et al. (2020) identified factors such as life disruption and uncertainty-reducing behavior as influencing intolerance of uncertainty. Therefore, future scale development should include items addressing these factors. The second is the unidentified factors contributing to perception differences. While this study highlights differences in perceptions between non-medical professionals and nurses, it does not address the factors contributing to these differences. Bridging this gap remains a challenge for future research, particularly in identifying effective interventions.

Aside from these two primary limitations, the study also has design constraints: This study employs a cross-sectional design, limiting the ability to draw causal inferences. Longitudinal studies tracking changes in risk perception over time or in response to educational interventions are necessary to elucidate the formation and evolution of these perceptions. There is also potential response bias. The reliance on self-reported data collected via an online survey introduces the possibility of response bias. Participants may have provided socially desirable answers rather than their true perceptions, potentially affecting the validity of the findings.

Furthermore, the study has selection bias concerns. The study sample consists solely of individuals with internet access who consented to participate, raising concerns about selection bias. This limitation may affect the representativeness of the sample and the generalizability of the results. The study has generalizability limitations. Focusing primarily on Japan, the study’s findings may be influenced by specific cultural norms and healthcare contexts. Lastly, it has theoretical perspective limitations. While providing a solid theoretical foundation on medical uncertainty, the study does not thoroughly explore how professional training influences risk tolerance. Integrating cognitive bias theories from psychology, such as the availability heuristic or loss aversion, could offer a more comprehensive explanation for these disparities.

Recognizing these limitations, future research should consider adopting longitudinal designs, incorporating qualitative interviews or experimental methods for additional validation, examining risk perception differences across various healthcare systems, and integrating cognitive bias theories to enhance the validity and applicability of the findings.

## Conclusions

This study quantitatively demonstrated significant differences between non-medical professionals and nurses in perceptions regarding risks during hospitalization, infection risks, and family accompaniment. These differences persisted even after adjusting for individual attributes and outpatient status. Non-medical professionals showed heightened sensitivity to risks, likely due to limited exposure to clinical uncertainties, whereas nurses demonstrated greater tolerance owing to their professional experiences.

Understanding these differences is critical for improving patient-centered care and ensuring effective communication between healthcare providers and the general public. The mechanisms underlying uncertainty distress, such as life disruptions and uncertainty-reducing behaviors, must be addressed to bridge the perceptual gap. Tailored educational programs for the public, alongside structured communication strategies within healthcare institutions, are essential for aligning risk perceptions and promoting interdisciplinary collaboration.

Moreover, the scale developed in this study provides a practical tool for assessing risk perception differences, which can guide interventions in clinical practice, health policy, and public education. Future research should explore the factors contributing to these perceptual gaps and develop strategies that foster mutual understanding, thereby enhancing trust, reducing unnecessary distress among patients and families, and ultimately contributing to safer, more responsive healthcare systems.

## Appendices

Study questionnaire

Q1. Please indicate your gender.

・Male

・Female

Q2. Please indicate your age.

(                   ) years old

Q3. Please indicate the prefecture where you live.

・Hokkaido　　　・Aomori　　　　　　  ・Iwate　　　・Miyagi　　　　 ・Akita　　　　　　　

・Yamagata　　　・Fukushima　　　　   ・Ibaraki　 　・Tochigi　　　　・Gunma　　　　

・Saitama　　　　・Chiba　　　　　　　・Tokyo　　  ・Kanagawa　　  ・Niigata

・Toyama　　    　・Ishikawa　　　　   　・Fukui　　   ・Yamanashi　　 ・Nagano　　　　　　　

・Gifu　　　　   　・Shizuoka　　　　　　・Aichi　　　・Mie　　　　　 ・Shiga

・Kyoto　　　    　・Osaka　　　　　　 　・Hyogo　　 ・Nara　　　　　・Wakayama

・Tottori　　　   　・Shimane　　　　　    ・Okayama　・Hiroshima　　  ・Yamaguchi　　　　

・Tokushima　　　・Kagawa　　　　　 　・Ehime　　  ・Kochi　　　　  ・Fukuoka

・Saga　　　　　   ・Nagasaki　　　　　・Kumamoto　・Oita　　　　　・Miyazaki

・Kagoshima　　　 ・Okinawa

Q4. Are you married?

・Single

・Married (including divorced/widowed)

Q5. Do you have children?

・Have children living together

・Have children not living together

Q6. Please indicate your occupation.

・Company employee　　　・Company owner

・Government employee / Teacher / Non-profit organization employee

・Self-employed (commercial/service industry)　・Agriculture, forestry, or fishery worker

・Physician　・Dentist　・Pharmacist　・Nurse　・Assistant nurse

・Public health nurse　　・Midwife　　・Physical therapist　・Occupational therapist

・Other healthcare professional　　・Lawyer / Tax accountant / Other legal or business professional

・Full-time homemaker　　　　 　・Student　・Unemployed　・Other occupation

Q7. Do you have a national qualification in the medical field?

・Yes

・No

Q8. Are you currently hospitalized or receiving outpatient treatment at a medical institution (hospital or clinic)?

・Yes

・No

Q9. Have you ever worked at a medical institution (hospital or clinic)?

・Yes

・No

Q10. What is the population size of your current residential area?

・Special wards (Tokyo 23 wards) or government-designated cities with a population of 500,000 or more

・Core cities with a population of 200,000 or more or special cities

・Cities with a population of 100,000 or more

・Cities with a population of less than 100,000

・Towns or villages with a population of less than 10,000

Q11. What is your highest level of education?

・Junior high school graduate

・High school graduate

・Vocational school graduate

・Junior college graduate

・University graduate

・Graduate school graduate

・Other (                    )

Q12. I do not think it is possible for me to contract an infectious disease from another patient during hospitalization. Please select the one that best matches your opinion.

・Strongly agree ・Quite agree ・Somewhat agree ・Neutral ・Somewhat disagree

・Quite disagree ・Strongly disagree

Q13. I do not think it is possible for me to develop bedsores on my buttocks or heels during hospitalization due to prolonged pressure from the bed or rails, causing redness, blisters, or wounds. Please select the one that best matches your opinion.

・Strongly agree ・Quite agree ・Somewhat agree ・Neutral ・Somewhat disagree

・Quite disagree ・Strongly disagree

Q14. I do not think it is possible for me to fall or slip from the bed during hospitalization. Please select the one that best matches your opinion.

・Strongly agree ・Quite agree ・Somewhat agree ・Neutral ・Somewhat disagree

・Quite disagree ・Strongly disagree

Q15. I do not think it is possible for me to become disoriented about time or place, behave unusually, or act in ways I normally would not during hospitalization. Please select the one that best matches your opinion.

・Strongly agree ・Quite agree ・Somewhat agree ・Neutral ・Somewhat disagree

・Quite disagree ・Strongly disagree

Q16. I think it is acceptable to use measures such as wearing mittens or restraining my hands to prevent me from unintentionally removing important IV tubes during hospitalization, even if it restricts my freedom of movement.  Please select the one that best matches your opinion.

・Strongly agree ・Quite agree ・Somewhat agree ・Neutral ・Somewhat disagree

・Quite disagree ・Strongly disagree

Q17. I believe it is natural for family members to accompany older patients during their hospitalization.

 Please select the one that best matches your opinion.

・Strongly agree ・Quite agree ・Somewhat agree ・Neutral ・Somewhat disagree

・Quite disagree ・Strongly disagree

Q18. I believe it is natural for family members to accompany children during their hospitalization. Please select the one that best matches your opinion. Please select the one that best matches your opinion.

・Strongly agree ・Quite agree ・Somewhat agree ・Neutral ・Somewhat disagree

・Quite disagree ・Strongly disagree
